# Immunohistochemical and molecular detection of the expression of FGF23 in phosphaturic mesenchymal tumors including the non-phosphaturic variant

**DOI:** 10.1186/s13000-016-0477-3

**Published:** 2016-03-09

**Authors:** Eisuke Shiba, Atsuji Matsuyama, Ryo Shibuya, Kei Yabuki, Hiroshi Harada, Mitsuhiro Nakamoto, Takahiko Kasai, Masanori Hisaoka

**Affiliations:** Department of Pathology and Oncology, School of Medicine, University of Occupational and Environmental Health, 1-1 Iseigaoka, Yahatanishi-ku, Kitakyushu, 807-8555 Japan; Kagoshima Occupational and Environmental Health Center, 4-96 Tokaicho, Kagoshima, 891-0115 Japan; National Hospital Organization Kinki-Chuo Chest Medical Center, 1180 Nagasonecho, Kita-ku, Sakai, Osaka 591-8025 Japan

**Keywords:** Phosphaturic mesenchymal tumor, Tumor-induced osteomalacia, Non-phosphaturic variant, Fibroblast growth factor 23, Immunohistochemistry, Polymerase chain reaction

## Abstract

**Background:**

Phosphaturic mesenchymal tumors (PMTs) are rare neoplasms that are often associated with tumor-induced osteomalacia (TIO) due to excessive serum levels of fibroblast growth factor 23 (FGF23). PMTs share overlapping histologic features with other types of tumors; thus, accurate pathological diagnosis may be challenging. We performed an immunohistochemical examination of FGF23 expression in PMTs and other types of tumors, together with pertinent molecular analyses.

**Methods:**

Seven PMTs (5 with TIO and 2 without TIO) and 46 other types of bone and soft tissue tumors were retrieved, and immunohistochemistry was performed using a commercially available anti-FGF23 antibody. In addition, FGF23 mRNA expression was detected by reverse transcription-polymerase chain reaction (RT-PCR), using RNA extracted from formalin-fixed, paraffin-embedded tissues.

**Results:**

Immunohistochemical analysis of FGF23 expression showed distinct, punctate staining in the cytoplasm in 5 PMTs with TIO, whereas FGF23 expression was negative in the 2 PMTs without TIO and the other 46 tumors. FGF23 mRNA expression was detected in all 4 PMTs examined, as well as in 1 chondromyxoid fibroma and 1 myxoid liposarcoma. The real-time RT-PCR data showed that the relative expression levels of the FGF23 mRNA tended to be higher in PMTs with TIO than in PMTs without TIO, or in the chondromyxoid fibroma specimen.

**Conclusions:**

Our data suggested that the feasibility of immunohistochemical detection of FGF23 may depend on the level of secreted FGF23 from tumor cells. Thus, immunohistochemistry for FGF23 is an useful diagnostic adjunct for PMT, although its utility appears to be limited in cases without TIO.

## Background

Tumor-induced osteomalacia (TIO) was previously described as involving various types of bone and soft tissue tumors such as hemangiopericytoma (an obsolete term, which is now referred to as solitary fibrous tumor), chondroblastoma, and osteosarcoma [1–5]. Recent findings have revealed that the majority of such cases encompasses a single entity with distinctive histological features, and this specific group of lesions has been designated as phosphaturic mesenchymal tumor (PMT) [1–3]. The formation of TIO in PMTs is thought to result from an excessive serum level of tumor-derived fibroblast growth factor 23 (FGF23) [3–5], which is an osteocyte-derived phosphaturic hormone that inhibits renal phosphate reabsorption and the production of 1,25(OH)_2_ vitamin D by reducing the activity of renal 1α-hydroxylase [6–8]. However, rare PMTs likely express other phosphaturic hormones (e.g., frizzled-related protein 4 and matrix extracellular phosphoglycoprotein) [9–11]. The minor fraction of PMTs that lack clinical evidence of TIO is referred to as the non-phosphaturic variant [3].

In 2009, Bahrami et al. [12] reported the diagnostic utility of detecting FGF23 transcripts in PMTs by the reverse transcription-polymerase chain reaction (RT-PCR) using formalin-fixed, paraffin-embedded (FFPE) tumor tissues. Although RT-PCR testing is highly sensitive, *FGF23* mRNA is considered to be structurally normal and could also be detected in non-PMT tumors, including aneurysmal bone cysts and chondromyxoid fibromas [12–14]. A more reliable diagnostic adjunct for routine pathology testing is required.

Here, we performed immunohistochemical staining for FGF23 expression in PMTs, with or without TIO, and other types of bone and soft tissue tumors using a commercially available anti-FGF23 antibody together with real-time RT-PCR, This approach allowed us to address differences in FGF23 expression between PMTs, with and without TIO.

## Methods

Archived specimens from 7 PMTs (5 with TIO and 2 without TIO) and 46 other bone and soft tissue tumors (6 chondromyxoid fibromas, 4 chondroblastomas, 4 chondrosarcomas, 1 extraskeletal mesenchymal chondrosarcoma, 5 osteosarcomas, 3 synovial sarcomas, 2 angiosarcomas, 2 clear cell sarcomas, 1 myxoid liposarcoma, 4 solitary fibrous tumors, 4 giant cell tumors of the bone, 6 giant cell tumors of the tendon sheath, and 4 aneurysmal bone cysts) were obtained from our institution. PMT diagnosis was made based on clinical information and morphological findings, including dirty or smudgy calcification, as determined by two pathologists (M.H. & A.M.). For immunohistochemical examinations, histological sections of FFPE tumor specimens were incubated with an anti-FGF23 monoclonal antibody (FG322-3, 1:500 dilution; Adipogen, San Diego, CA, USA) at room temperature for 24 h after epitope retrieval in ethylenediaminetetraacetic acid buffer (pH 8.0) using a pressure cooker, followed by treatment with 3 % hydrogen peroxide for 10 min. Immunostaining was accomplished by incubation with a labeled polymeric secondary antibody (Histofine Simple stain MAX PO, Nichirei, Tokyo, Japan). Diaminobenzidine solution was used for visualization, followed by nuclear counterstaining with hematoxylin. As described by Nelson et al. [15] and Houang et al. [16], distinct “dot-like” cytoplasmic staining of FGF23 was considered to represent a positive result, whereas diffuse cytoplasmic and nuclear staining were interpreted as non-specific findings.

For molecular detection of the *FGF23* gene transcript, total RNA was extracted from FFPE tissues using the TRIzol reagent (Invitrogen, Carlsbad, CA, USA) and reverse transcribed into cDNA. RT-PCR-based analysis of the *FGF23* transcripts was performed using 3 different sets of primers designed by Bahrami et al. [12]. The transcripts of reference genes (phosphoglycerate kinase and porphobilinogen deaminase) were amplified along with *FGF23* as quality controls. Quantitative analysis of *FGF23* mRNA expression was performed by real-time RT-PCR analysis using a TaqMan Gene Expression Assay (Applied Biosystems, Foster City, CA, USA), according to the manufacturer’s instructions. Briefly, 20-μl PCR reaction mixtures containing 1× TaqMan Gene Expression Master Mix, 1× TaqMan Gene Expression Assay, and the reverse transcription products were incubated at 95 °C for 10 min, followed by 40 cycles at 95 °C for 15 s and at 60 °C for 1 min. Standard curves were generated to quantitate the data. *FGF23* mRNA expression levels were normalized to that of the glyceraldehyde 3-phosphate dehydrogenase (*GAPDH*) gene, as an endogenous control.

The study design was approved by an ethics review board of the University of Occupational and Environmental Health (H25-169).

## Results and discussion

**Fig. 1:**
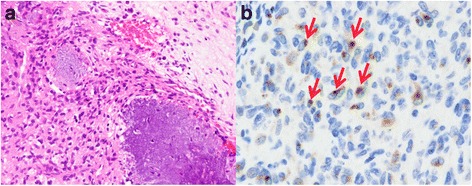
PMT with TIO. **a**: The tumor was characterized by the proliferation of bland spindle or oval cells, with grungy calcification. **b**: By immunohistochemical staining, FG322-3 expression showed a distinct, dot-like staining pattern (*arrows*)

**Fig. 2:**
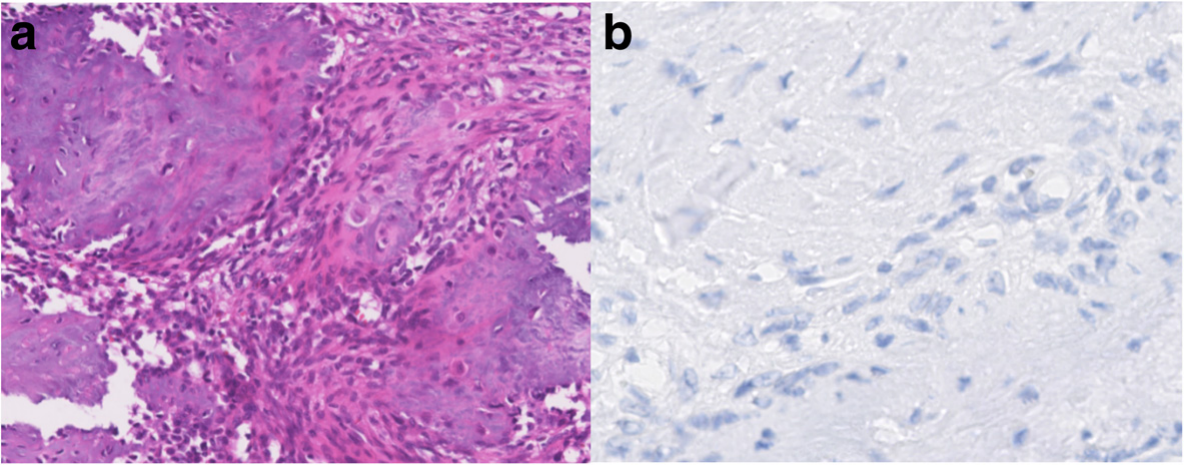
PMT without TIO. **a**: The tumor was characterized by the proliferation of bland spindle cells, with grungy calcification. **b**: Immunohistochemical staining showed that the tumor cells were negative for FG322-3

**Fig. 3:**
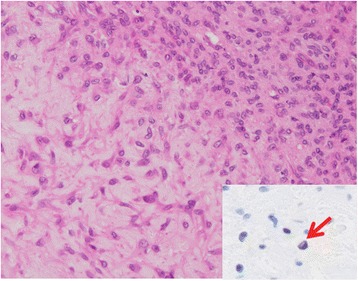
Chondromyxoid fibroma. Inset: Weak nuclear staining of FG322-3 was noted in a small proportion of the tumor cells (*arrow*)

**Fig. 4:**
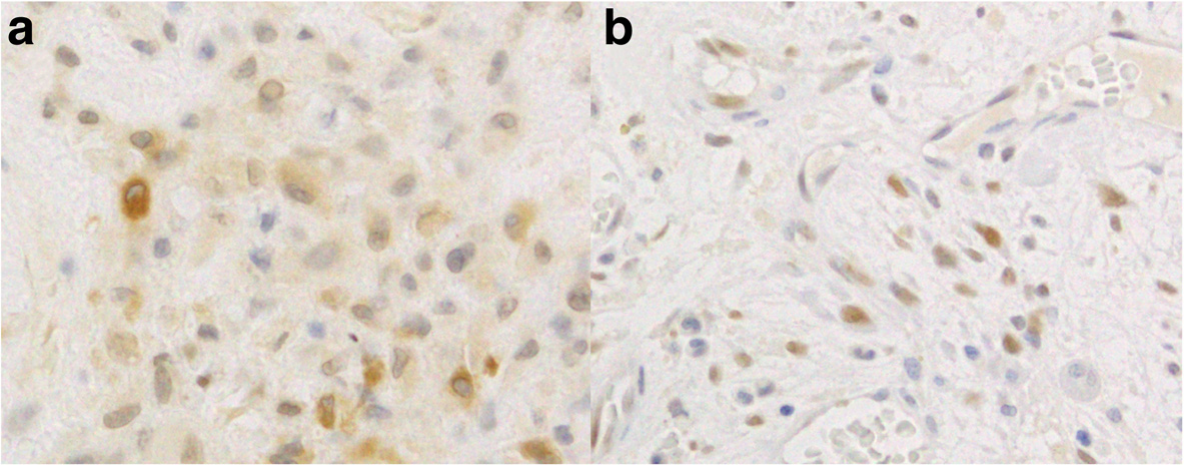
Non-specific FG322-3 immunostaining observed using a high antibody concentration. **a**: Diffuse cytoplasmic staining in a chondromyxoid fibroma. **b**: Nuclear staining in an aneurysmal bone cyst

The results of our immunohistochemistry analysis of FGF23 expression in the tumor specimens examined are summarized in Table 1. Five of 7 (71.4 %) PMTs were positive for FG322-3, and showed a distinct, punctate intracytoplasmic and perinuclear staining pattern in 5–20 % of the tumor cells (Fig. 1). Most positive cells were oval or spindle-shaped and tended to reside in densely populated cellular areas, rather than hypocellular calcified areas. All 5 of the FG322-3-positive PMTs had TIO. In contrast, no FG322-3-positive cells were observed among the 2 PMTs without TIO (Fig. 2) or the 46 non-PMT tumors, although non-specific nuclear and/or diffuse cytoplasmic staining was noted in 9 cases (Figs. 3 and 4).

**Table 1:** Immunohistochemical results of FGF23

Diagnosis	Positive
Phosphaturic mesenchymal tumor	5/7
Chondromyxoid fibroma	0/6
Chondroblastoma	0/4
Chondrosarcoma	0/4
Extraskeletal mesenchymal chondrosarcoma	0/1
Osteosarcoma	0/5
Synovial sarcoma	0/3
Angiosarcoma	0/2
Clear cell sarcoma	0/2
Myxoid liposarcoma	0/1
Solitary fibrous tumor	0/4
Giant cell tumor of bone	0/4
Giant cell tumor of tendon sheath	0/6
Aneurysmal bone cyst	0/4

**Fig. 5:**
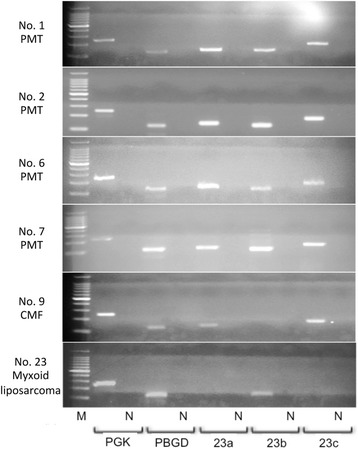
RT-PCR analysis of *FGF23* transcripts. All 3 *FGF23* transcripts (23a, 140 bp; 23b, 125 bp; 23c, 175 bp) were amplified in PMTs (with TIO: case Nos. 1, 2, without TIO: case Nos. 6, 7), whereas only 2 or 1 of the 3 transcripts were detected in the chondromyxoid fibroma sample (case No. 9) or the myxoid liposarcoma sample (case No. 23), respectively. M, 100-bp DNA ladder; N, negative control; PGK, phosphoglycerokinase; PBGD, porphobilinogen deaminase; CMF, chondromyxoid fibroma

**Fig. 6:**
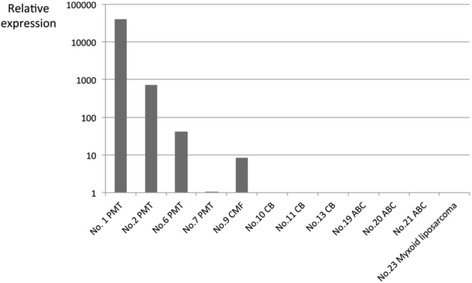
The relative expression levels of *FGF23* in PMTs, as determined by real-time RT-PCR analysis. The expression level of *FGF23* was higher in PMTs with TIO (case Nos. 1 and 2) than in PMTs without TIO (case Nos. 6 and 7) or chondromyxoid fibroma (case No. 9). The *FGF23* gene transcript was not identified in the other tumors examined, including myxoid liposarcoma (case No. 23). Expression levels were normalized to *GAPDH* as an internal control

Results of the cases evaluated by a RT-PCR analysis are summarized in Table 2. During RT-PCR analysis, *FGF23* gene transcripts were identified in all 4 PMTs examined, 2 of which were associated with TIO (Fig. 5). Only 2 of the 3 *FGF23* transcripts examined were detected in a chondromyxoid fibroma, and 1 of the 3 studied transcripts was identified in a myxoid liposarcoma (Fig. 5). No *FGF23* transcript was amplified in the other tumors examined. Analysis of our real-time RT-PCR data showed that the relative expression levels of the *FGF23* gene transcripts tended to be higher in PMTs with TIO than those without TIO, or in chondromyxoid fibroma samples (Fig. 6). The *FGF23* gene transcript was not amplified in the other tumor specimens examined, including the myxoid liposarcoma specimen (Fig. 6).

**Table 2:** Results of the cases evaluated by a RT-PCR analysis

No	Age (y)/sex	Site	Diagnosis	TIO	IHC	RT-PCR
						FGF23a/b/c
1	35/F	Popliteal region	Phosphaturic mesenchymal tumor	+	+	+/+/+
2	59/F	Groin	Phosphaturic mesenchymal tumor	+	+	+/+/+
3	59/F	Nasal cavity	Phosphaturic mesenchymal tumor	+	+	NA
4	61/M	Lower leg	Phosphaturic mesenchymal tumor	+	+	NA
5	46/F	Nasal cavity	Phosphaturic mesenchymal tumor	+	+	NA
6	38/F	Foot	Phosphaturic mesenchymal tumor	-	-	+/+/+
7	43/M	Radius	Phosphaturic mesenchymal tumor	-	-	+/+/+
8	57/M	Distal phalanx	Chondromyxoid fibroma		-	NA
9	30/M	Fibula	Chondromyxoid fibroma		-	+/−/+
10	23/M	Patella	Chondroblastoma		-	−/−/-
11	15/M	Humerus	Chondroblastoma		-	−/−/-
12	26/M	Calcaneus	Chondroblastoma		-	−/−/-
13	57/M	Tibia	Chondroblastoma		-	−/−/-
14	74/F	Middle finger	Giant cell tumor of tendon sheath		-	−/−/-
15	66/M	Index finger	Giant cell tumor of tendon sheath		-	−/−/-
16	51/F	Wrist	Giant cell tumor of tendon sheath		-	−/−/-
17	62/F	Femur	Giant cell tumor of bone		-	−/−/-
18	39/M	Femur	Giant cell tumor of bone		-	−/−/-
19	70/F	Femur	Aneurysmal bone cyst		-	−/−/-
20	74/F	Femur	Aneurysmal bone cyst		-	−/−/-
21	71/F	Vertebra	Aneurysmal bone cyst		-	−/−/-
22	20/M	Thigh	Extraskeletal mesenchymal chondrosarcoma		-	−/−/-
23	65/F	Lower leg	Myxoid liposarcoma		-	−/+/−

*IHC* immunohistochemistry, *NA* not available, *RT-PCR* reverse transcription-polymerase chain reaction, *TIO* tumor-induced osteomalacia

PMT is a rare neoplasm of soft tissues and bones that arises predominantly in middle-aged adults and generally has a benign clinical course [3–5]. The characteristic morphological features of PMTs include bland spindle cell cytomorphology, a low mitotic activity, grungy or flocculent calcification, and a myxoid or myxochondroid stroma [3–5]. Osteoclast-like giant cells, microcystic changes, an osteoid matrix, hemangiopericytomatous blood vessels, and a mature adipose-tissue component may be present in some cases [3–5]. However, some PMTs may contain areas of high-grade sarcoma resembling undifferentiated pleomorphic sarcoma or fibrosarcoma and often behave in an aggressive manner. In addition, histologically benign PMTs may also metastasize [17–20]. An accurate diagnosis of PMT is often difficult due to histological heterogeneity, and differential diagnoses include many other types of tumors with some similar morphologic features, such as chondromyxoid fibroma, chondroblastoma, aneurysmal bone cyst, and osteosarcoma.

In this study, we found that the immunohistochemical expression of FGF23 was highly specific for PMTs with TIO (100 %). The sensitivity was 71.4 % in our overall series of PMTs tested, and 100 % in the phosphaturic variant. Although the number of examined tumor samples was small, our data suggest that FGF23 is a useful immunohistochemical marker for PMT with TIO. It is important to note that only the punctate staining pattern was considered to be positive, similar to previous studies using other anti-FGF23 antibody clones [15, 16]. Diffuse cytoplasmic and nuclear staining are occasionally encountered in non-PMT tumors or normal tissues at inappropriately high antibody concentrations, whereas such staining patterns are inconspicuous or absent at low concentrations. In addition, no *FGF23* gene transcript was detected by RT-PCR analysis in non-PMT tumors displaying these staining patterns. Thus, the perinuclear, dot-like staining pattern is reliable phenotypic characteristic of FGF23 expression. Similar perinuclear staining patterns of other markers have been observed during the immunohistochemical analyses of several other tumor types, including Merkel cell carcinoma (cytokeratin 20) [21], desmoplastic small round cell tumors (desmin) [22], and Hodgkin’s lymphoma (CD30) [23]. In these tumors, the unique staining patterns are considered to result from clumping of intermediate filaments [21, 22], or the accumulation of protein precursors in the Golgi area. [23]. In PMTs, the punctate staining pattern may result from an accumulation of highly expressed FGF23 precursor molecules in the Golgi apparatus, although further investigations are needed to address the mechanisms underlying the unique intracellular localization pattern of FGF23.

Immunohistochemical examination of FGF23 expression in tumors using a commercially available antibody has been limited thus far [16]. Folpe et al. [3] reported that not only PMTs with TIO, but also PMTs without TIO and some other tumors were immunohistochemically positive for FGF23, using a polyclonal antibody. They scored diffuse cytoplasmic staining as positive and concluded that this staining was not specific. Houang et al. [16] reported that 14 PMTs (all with TIO) and 2 of 40 non-PMT tumors in their series were positive for FGF23 using a different commercially available antibody (polyclonal, 1:100 dilution; Immutopics Inc., San Clemente, CA, USA). The cytoplasmic dot-like expression pattern and the distribution of positive tumor cells observed in their study appeared similar to our results. Although single cases of aneurysmal bone cyst and osteosarcoma were also positive for FGF23 in their study, it is difficult to conclude that these tumors are unequivocally different from PMTs without TIO, due to their potential morphological overlap.

By RT-PCR analysis, *FGF23* gene transcripts were identified not only in the 4 PMTs examined, but also in 1 chondromyxoid fibroma and 1 myxoid liposarcoma. However, no *FGF23* gene transcripts were detected in the myxoid liposarcoma sample by real-time RT-PCR. The difference of the primer sequences used in either analysis might contribute to these incongruous results. Because only 1 of the 3 targeted transcripts was detected in the myxoid liposarcoma sample by RT-PCR, a potentially non-functioning splicing variant of *FGF23* might have been amplified. Semi-quantitative analysis of the *FGF23* gene transcript by real-time RT-PCR showed that the relative expression level tended to be higher in PMTs with TIO than in PMTs without TIO. Tumor cells in the non-phosphaturic variant of PMT may produce *FGF23* at a low level. The immunohistochemical expression of FGF23, as well as the clinical manifestation of TIO, may be dependent on the level of secreted FGF23 by tumor cells. The expression level of the *FGF23* gene transcript in the chondromyxoid fibroma was also lower than observed in PMTs with TIO; however, it was comparable to PMTs without TIO. These results raise a concern as to whether chondromyxoid fibroma can be completely distinguished from the non-phosphaturic variant of PMT, because it is occasionally challenging to differentiate chondromyxoid fibroma from PMT [24, 25]. Chondromyxoid fibromas typically show a distinct lobular architecture and rarely contain dilated blood vessels. However, the clinical information of TIO may be overlooked, and the histopathological distinction of these tumors may be somewhat arbitrary. Recently, the *FN1-FGFR1* fusion gene was identified in PMTs using next-generation RNA sequencing [26], and upregulation of metabotropic glutamate receptor 1 (GRM1) expression through gene fusion and promoter swapping was detected in chondromyxoid fibroma [27]. These distinct molecular profiles may be helpful to differentiate both entities or to diagnose the non-phosphaturic variant of PMT.

## Conclusions

The immunohistochemical expression of FGF23 in PMTs may depend on the level of secreted FGF23 from tumor cells. Thus, immunohistochemical staining for FGF23 may serve as a diagnostic adjunct of PMTs, particularly those with TIO, although further investigations using a greater number of clinical specimens are necessary.

## Abbreviations

FGF23: fibroblast growth factor 23
PMT: phosphaturic mesenchymal tumor
RT-PCR: reverse transcription-polymerase chain reaction
TIO: tumor-induced osteomalacia
